# 

**Published:** 1890-10

**Authors:** 


At the time of going to press the signatnres of Drs. Clopton and Harrison, two of the Committee on Legislation, had not been received to the Circular Letter published in this issue, but the 6000 reprints will bear the signatures of the full committee.—ED.

